# vSEMERA: pilot project assessing health profession students’ experiences in an international virtual research program

**DOI:** 10.1186/s12909-024-05528-6

**Published:** 2024-06-01

**Authors:** Laura Bell, Eliana Lemos, Jan Krimphove, Stephanie Kaiser, Cristina Guerra-Giraldez, Martin Lemos

**Affiliations:** 1 Audiovisual Media Center, Medical Faculty, RWTH Aachen University, Aachen, Germany; 2 Dean of Studies and Teaching Office, RWTH Aachen University, Aachen, Germany; 3 Centro Universitário Christus (Unichristus), Fortaleza, Brazil; 4 Institute for History, Theory and Ethics of Medicine, Medical Faculty, RWTH Aachen University, Aachen, Germany; 5 Mauthausen Memorial, Vienna, Austria; 6 Institutional Affairs and Internationalization, School of Science and Engeneering, Universidad Peruana Cayetano Heredia, Lima, Peru

**Keywords:** Virtual exchange, Health profession, Scientific skills training, Medical education

## Abstract

**Background:**

The “Virtual Semester for Medical Research Aachen” (vSEMERA) is an international, interdisciplinary, virtual education program developed for health profession students. The first edition (2021) was hosted by the Medical Faculty of RWTH Aachen University (Germany) in cooperation with Centro Universitário Christus (Brazil) and Universidad Peruana Cayetano Heredia (Peru). The primary aim of the 12-weeks program was to provide students with skills in health science research and prepare them for scientific career paths.

**Methods:**

vSEMERA was built on a virtual learning platform, the “vSEMERA-Campus”, designed to foster students’ learning process and social interactions. Maximum flexibility was offered through synchronous and asynchronous teaching, enabling participants to join via any device from any part of the Globe alongside their regular studies. For the program’s first edition (September - November 2021), health profession students from Germany, Brazil, Peru, Spain, and Italy filled all 30 available spots. Satisfaction, quality of the program and courses offered, as well as perceived learning outcomes, were examined using questionnaires throughout and at the end of the program.

**Results:**

The program received a rating of 4.38 out of 5 stars. While it met most expectations (4.29 out of 5), participants were unable to attend as many courses as intended (2.81 out of 5), mainly due to scheduling conflicts with the home university schedule (46%), internships (23%), and general timing issues (31%). Participants acknowledged considerable improvements in their scientific skills, English language skills, confidence in scientific project management, research career progression, and enthusiasm for a scientific career.

**Conclusions:**

vSEMERA represents a promising example of an online international learning and exchange program using pedagogical and technological elements of virtual collaboration and teaching. In addition to advancing future vSEMERA editions, our results may offer insights for similar projects that address the targeted integration of scientific research education into an international, digital learning environment.

## Introduction

Knowledge and skills related to scientific and research methods are critical to conducting research, pursuing a research career, and ensuring competence as a health professional [1]. The World Federation for Medical Education (WFME) enforces within the *‘Global Standards for Quality Improvement in Medical Education’* that medical education *must* teach the basic knowledge (principles of the scientific method, including analytical and critical thinking, medical research methods, and evidence-based medicine) as well as *should* include elements of original or advanced research. These aspects are also mapped in the German National Competence-Based Learning Objectives Catalog for Medicine (NKLM, VIII.1.) [2]. Yet, while scientific research is expanding and the importance of research knowledge and skills is known, the number of clinicians and healthcare professionals participating in research appears to be shrinking [1, 3–5]. To identify potential barriers to the development of research skills and to propose strategies to overcome and ultimately counteract this development, the Association for Medical Education in Europe (AMEE) formulated a guidance paper for the education of medical students [1]. One critical issue identified in the guidance paper is that in addition to a demanding medical curriculum, opportunities, and resources to develop the necessary research skills are limited [1]. A recent scoping review explores curriculum initiatives aimed at solving the issue of the shrinking number of clinicians and healthcare professionals participating in research by improving the research experience for medical students [6]. For the reported non-intercalated programs, most of the research initiatives were elective research projects (e.g., summer research projects), while for the reported intercalated programs, the research initiatives were related to the bachelor’s or PhD programs [6]. While all research initiatives show overall benefits in developing a research career and self-efficacy, factors that make research training in or alongside medical training difficult are the cost of time, availability of mentors, and student motivation. In addition, schools’ resources might vary. While medical students in high- and low-income countries appear equally willing to conduct research [7], Carberry et al. [6] noted that fewer studies on research training were conducted in low- and middle-income countries. Low-income countries often have limited resources to finance equipment, research, and train personnel [8].

International virtual exchange (IVE) is a structured, technology-mediated educational approach [9]. It has the potential to reduce the burden of limited resources and personnel not only in low-income countries, but also internationally, while promoting international social and scientific collaboration between future medics and medical researchers directly from their home country. Concomitantly, IVE aligns with the rapidly evolving healthcare system and digitization [10]. The need for novel, alternative online teaching methods in medical education became particularly evident during the COVID-19 pandemic [11–15]. While online learning initially used to be a mere supplement to classroom learning, it has over the years become a successful tool that can offer a range of services from distance learning to computer-assisted virtual patient simulations [16] and the effects on medical training appear promising (see, e.g [16–18]). Importantly, although education of medical degrees might vary across the globe, medical core competencies should be internationally applicable. Thus, international online training programs and IVE could benefit medical research worldwide. In addition, global learning offers the opportunity to encourage students to explore other cultures, life experiences and world views [18]. Yet, while the first steps have been taken towards international medical training programs (e.g., the CoBaTrICE Collaboration [19], which aims to identify core competencies in adult intensive care to develop an international training program), there are, to the best of our knowledge, only a few available international online training programs for medical education [20–22] and no online training programs for research skills for medical students.

Given the potential benefits of IVE, the first edition of the “virtual Semester for Medical Research Aachen” (vSEMERA) was developed as an interdisciplinary, IVE program for health profession students to train their research skills. It was hosted by the Medical Faculty of the RWTH Aachen University (Germany) in cooperation with Centro Universitário Christus (Brazil) and Universidad Peruana Cayetano Heredia (Peru). The primary aim of the current manuscript is to highlight and evaluate the first edition of the IVE program in 2021. To assess the quality of the program and the learning outcomes, students’ self-reports were examined.

## Materials and methods

### Ethical approval

The study was approved by the Ethics Committee of the Medical Faculty of RWTH Aachen University (EK 464/21). Informed consent was obtained from all students before participation. All questionnaires were answered anonymously, and evaluations were voluntary.

### The IVE program

vSEMERA is a 12 week-long multi-institutional and interdisciplinary IVE program that builds on a virtual learning platform “vSEMERA Campus”, an intercultural social program and keynote lectures from experts in the field of health care and empirical research. Participants learn how to conduct research and apply scientific methods. The program’s first edition took place from September to November 2021 with 30 health profession students from Brazil, Germany, Italy, Peru, and Spain, and was funded by the Federal Ministry of Education and Research (BMBF) and the German Academic Exchange Service (DAAD). Students were required to submit a CV and a letter of motivation and participate in a research project in health science. After a thorough review and evaluation of all applications, 30 students were selected from 64 initial candidates. With the successful completion of the program, participants received a transcript of records with credits (15 ECTS) and an attachment listing all courses. The workload of vSEMERA of 15 ECTS equals approximately 450 h. Additionally, the local students received a certificate of completion, in compliance with the mandatory elective courses. vSEMERA is linked to the medical curriculum of RWTH Aachen University within the mandatory electives.

### vSEMERA Campus

**Fig. 1:**
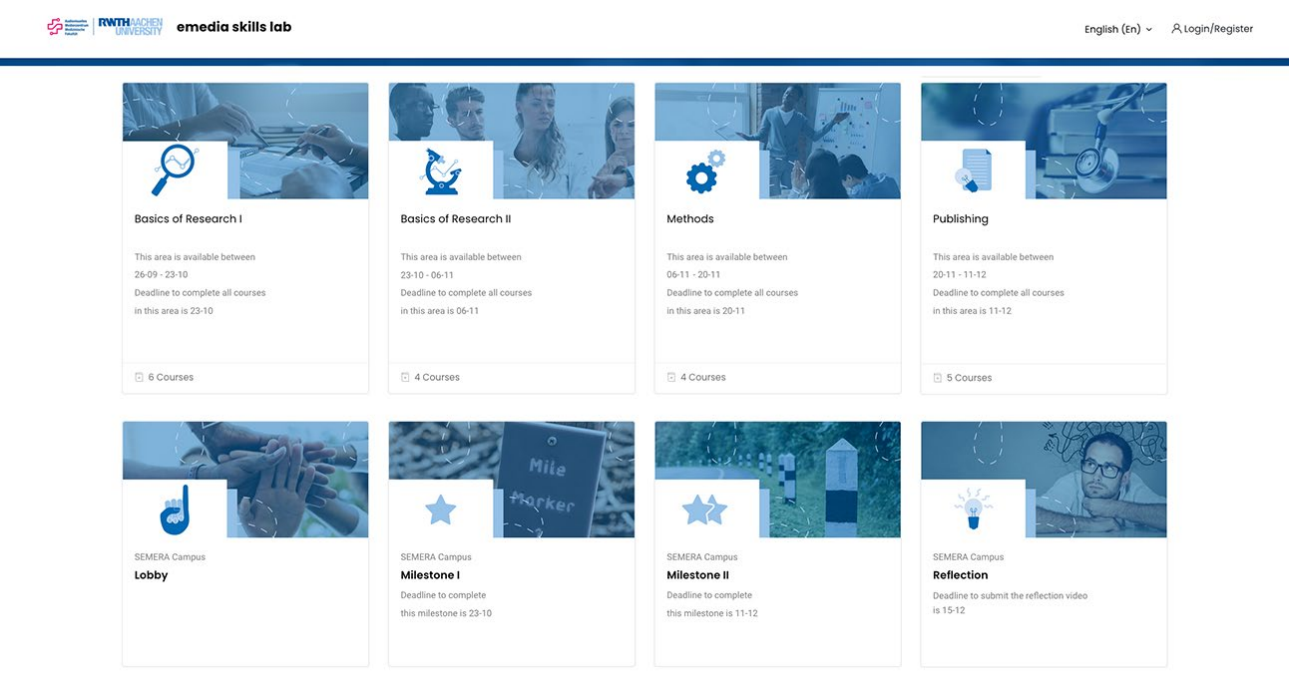
Main menu of the digital learning platform vSEMERA campus

vSEMERA Campus is the digital learning platform for participants of the program. This specially developed module is based on the learning management system *Moodle* (https://moodle.org). All courses of the program were mapped here and could be accessed by the participants at any time and on different devices. The structure of the courses within the vSEMERA campus was standardized so that lecturers and students could easily navigate through all of them. Nevertheless, lecturers could adapt their courses individually. That is, the digital “course rooms” could be equipped with a zoom meeting, lecture recordings, course materials, assessments, and a forum. See Fig. 1 for a screenshot of the digital learning platform.

#### Courses

A total of 26 courses were offered within the program. Course topics ranged from organizational and vSEMERA-specific topics, i.e., an introduction to vSEMERA and vSEMERA Campus, over digital and research skills to (doctoral) thesis-related topics, such as conducting a research project, statistics, giving a presentation and publishing a paper. For a complete list of all courses, see Table 1. The courses and course content were suggested by experts in the field, aligned with the Longitudinal Scientific Curriculum of the Medical Faculty of the RWTH Aachen University. The final curriculum was discussed with the curriculum developer in Aachen and then considered core research skills for a 12-week program. Participation in almost all courses was voluntary, and participants could decide which courses were relevant and interesting. Only three courses (“*How to write a research proposal”*, “*How to run a research project”* and “*How to design a poster and present it”*) that were relevant for reaching the program’s milestones were mandatory for the students. Maximum flexibility was allowed through synchronous as well as asynchronous teaching. On average, there were about 3 courses per week.

**Table 1:** Course list of vSEMERA

Group	Courses
Scientific Basics	• Current state of the scientific method • Design thinking • Digital skills • Good scientific practice • How to choose a doctoral thesis • How to read and evaluate scientific papers • How to run a research project • How to write a research proposal • Scientific Integrity • Research Data Management
Scientific Methods	• A clinician’s guide to biomedical statistics using large, population-based datasets • Cell culture models in cancer research • General laboratory practice for translational medicine • (Systematic) Literature research • Research on High Altitude physiology • Statistical competences
Publications	• Creation of tables and figures • Good presentation techniques • How scientific is nutrition, really? • How to design a poster and present it • How to gain (social media) visibility • Patents and standards • Scientific publishing from the viewpoint of a journal editor • Submission and publishing process of a scientific paper
Keynotes	• Career paths in science and research / career path of a scientist • Public health research in Peru • Research Conditions in the Global North and Global South • The use of psychedelic drugs from the Amazon rainforest in psychiatric therapeutics
vSEMERA specific courses	• Diversity icebreaker • How to use vSEMERA Campus • Introduction to vSEMERA • Presentation of the Brazilian Medical System • Presentation of the German Medical System • Presentation of the Peruvian Medical System

### Milestones

To successfully complete the program, students had to pass two mandatory milestones. To reach milestone I students had to write a scientific abstract (1,500 words) about their research project. The abstract was rated by the lecturers as either sufficient or insufficient. To reach milestone II students had to create a scientific poster and present it. The poster was evaluated during the hybrid poster walk, see below.

### Keynotes

Throughout the program, a series of four keynotes by invited experts in their fields was offered to the participants as well as external participants outside the program in the form of virtual lectures in *Zoom* (Zoom Video Communications Inc., San Jose, CA). In 2021, the invited experts came from the Peruvian University Cayetano Heredia in Lima (Peru), and the Federal University of Rio Grande do Norte in Natal (Brazil), see Table 2. The keynote topics were suggested and discussed by the vSEMERA organizational and scientific team (Aachen, Lima, Fortaleza).

**Table 2:** Overview of the keynotes

Keynote		Title	Lecturer	Online recording
1		Career paths in science and research /career path of a scientist	Professor Dr. Dionicia Gamboa, Universidad Peruana Cayetano Heredia (Lima, Peru)	No recording available
2		Research conditions in the Global North and Global South	Dr. Cristina Guerra Giraldez, Universidad Peruana Cayetano Heredia (Lima, Peru)	No recording available
3		Public Health Research in Peru	Dr. Ernesto Gozzer, Universidad Peruana Cayetano Heredia (Lima, Peru)	https://youtu.be/ls7rvn9V_R0
4		The use of psychedelic drugs from the Amazon rainforest in psychiatric therapeutics	Dr. Fernanda Palhano and Professor Dr. Draulio Barros de Araujo, Universidade Federal do Rio Grande do Norte (Natal, Brazil)	https://youtu.be/5HuWU3xLPO4

### Social Program

To foster scientific as well as social networking across the participants of the different universities, a social program was offered to the students. Within the social program, students participated in speed dating-type sessions, presented their home university, and upon students’ wishes also explored the history of each country together in virtual meetings hosted in *Zoom* (Zoom Video Communications Inc., San Jose, CA).

### Blended Staff Mobility

Next to the exchange on student level, the program also fostered the exchange and cooperation between participating lecturers from all universities and across various disciplines. Thanks to the available funding, the staff from the RWTH Aachen University was able to visit the Centro Universitário Christus (Brazil) as well as the Universidad Peruana Cayetano Heredia (Peru) to explore the research facilities and discuss cooperations as well as vSEMERA specific aspects. An impression of the visits and the two Universities in Brazil and Peru can be obtained in the following video, which was first presented during the closing event during the hybrid poster walk: https://www.youtube.com/watch?v=pgiw9-Ps74o. A Team from the Centro Universitário Christus (Brazil) and from the Universidad Peruana Cayetano Heredia (Peru) was able to attend the farewell week in Aachen. This visit was of great importance for networking and cooperation, as the employees had the opportunity to visit other departments, clinics and faculties. The visits were arranged by the hosting faculty, according to the wishes and interests of the visiting staff.

### Farewell Week and Poster Presentations

**Fig. 2:**
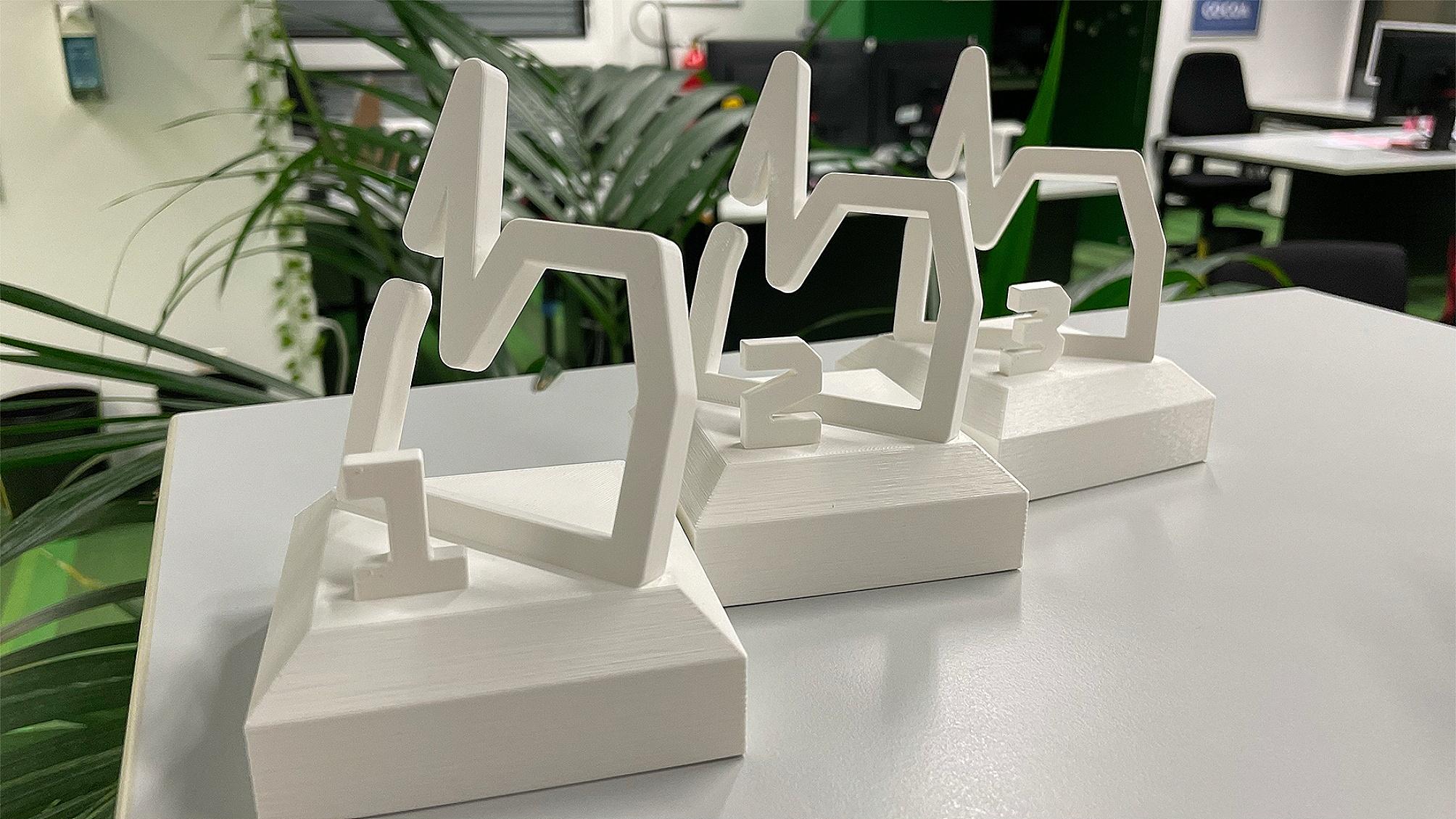
Impressions of the farewell week showing the vSEMERA trophies for the best posters

The program ended with the farewell week, which was organized as a hybrid event. Thanks to the DAAD funding, 10 out of the 30 students (five from Brazil und five from Peru) were able to travel to Aachen, Germany, for the farewell week. During the entire week, the students had the opportunity to get a first-hand look at a wide range of institutes and research departments at the RWTH Aachen University and the RWTH Aachen University Hospital. The remaining students were able to participate online on the final day (Poster Walk) of the farewell week. During the last event, students presented their scientific projects that they had been working on in the form of a poster presentation (part of milestone II). Students’ posters and poster presentations were rated by a jury. The jury consisted of seven lecturers from the three collaborating universities. Ratings concerned the overall impression (two items) and poster design/layout (three items), the content (three items) as well as the verbal poster presentation (five items). All items were rated on a 6-point Likert Scale (0 - gross inaccuracies to 5 - very good, no inaccuracies) (Table 3). The farewell week ended with an award ceremony for the three best posters and a farewell dinner for all students on site (Fig. 2).

**Table 3:** Poster rating criteria

Topic	Criteria
Appearance/overall impression	At first glance, the poster makes overall a professional (and aesthetic) impression. Visually, the ratio of graphic elements and text is balanced, and they are spatially clearly separated.
	The typographic elements (text alignment, highlighting, font sizes, etc.) are well coordinated and contribute to an appealing overall image.
Layout	The poster is divided into clearly recognizable segments.
	The graphic elements are well designed, functional and understandable. Similar objects are formatted in the same way. Objects and text fields are aligned.
	The amount of information conveyed on the poster is appropriate (not too much, not too little).
Content	The contents of the poster are scientifically sound.
	The text portions of the poster are written in a way that is understandable and appropriate for the audience.
	The language used is error-free.
Presentation	The linguistic expression is scientifically appropriate (not colloquial).
	It was performed at an appropriate pace, loudly and clearly.
	The presentation is well structured.
	The derivation of the research question is comprehensible and justified. A reference of the results to the research question was made.
	Questions were answered confidently.

### Questionnaires

Access to all questionnaires was provided via links that were available on the vSEMERA campus and were additionally sent to students by e-mail. The questionnaires could be filled in online through *LimeSurvey* (LimeSurvey: An Open-Source survey tool / LimeSurvey GmbH, Hamburg, Germany. URL http://www.limesurvey.org). The questionnaires were answered anonymously, using an identifier to link answers across questionnaires. Two kinds of questionnaires were utilized to evaluate (a) participants’ satisfaction with the overall program and its potential in training the participants research skills and (b) a questionnaire to evaluate each of the courses selectively. An overview of the items can be found in Table 4. Items were rated on a 5-point Likert Scale (0 - completely disagree, 5 - completely agree). In addition, open ended questions were asked about valued aspects as well as possible improvements of the program and the courses.

**Table 4:** Items of the overall program and course specific questionnaires

Questionnaire	Topic	Item
Overall program questionnaire	Overall	How many stars would you award to the entire vSEMERA program (the more stars the better you enjoyed and valued the program)?
		My expectations regarding the program were fully met.
		The program was too demanding.
		The program didn’t challenge me enough.
	Subjects/Courses of the program	All subject areas were sufficiently addressed.
		The contents conveyed were well related to practice.
		The topics of courses were well matched.
		A lot of course content was overlapping.
		Subjects were treated too superficially. If you agree, please name which subjects were treated superficially:
		Some subjects were irrelevant. If you agree, please name which subjects were irrelevant:
	Research benefit of the program	The program helped me a lot in improving my scientific skills.
		Through vSEMERA I have been able to improve my English significantly.
		The program was helpful in advancing my research career.
		The program motivated me to pursue a scientific career.
		The knowledge I gained from vSEMERA will also benefit my future activities and tasks.
		I think that the knowledge acquired in the program will be outdated or obsolete in the medium to long term.
	Ease of scheduling	The breaks between courses were sufficient.
		The course dates were generally adhered to and changes were sufficiently announced.
		It makes sense to take a semester off to participate in the program.
		Participating in the vSEMERA program alongside regular studies was no problem.
	Group size	There were too few participants. If you agree, what would be the ideal number of participants?
	Attendance	I was able to attend as many courses and lectures as I thought I would. If not, why were you not able to attend as many courses and lectures as you thought you would or as you would have liked to attend? What could be improved to increase the attendance?
	Feedback	What did you like most about the program? Please comment. What didn’t you like about the program?/ *What* could be improved? Please comment.
Course-related questionnaire	Course specific ratings	How many stars would you award this course (the more stars the better you liked the course)?
	Feedback	Which aspects of the course did you particularly appreciate? Which aspects of the course could be improved?

### Sample

Four students from RWTH Aachen University (Germany), two students from Spain (Universidad de Sevilla and Universidad de Castilla), four from Italy (Università degli Studi di Napoli Federico II and Università degli Studi di Sassari), ten students from Centro Universitário Christus (Brazil) and ten students from Universidad Peruana Cayetano Heredia (Peru) participated in the program. 56.67% of the participants were female. Students’ backgrounds ranged from medicine (*n* = 22), biology (*n* = 1), biomedicine (*n* = 5), computer science (*n* = 1), psychology (*n* = 1), and occupational therapy (*n* = 1). Of the 30 students, 21 filled in the questionnaire about satisfaction with the overall and social program and 28 students filled in course evaluations. Seven students dropped out before the end of the program and did not attend the whole program.

### Analyses

Descriptive analyses were carried out in R [23]. The *ggplot2* [24] and *forestplot* [25] packages were used for data visualization.

## Results

**Fig. 3:**
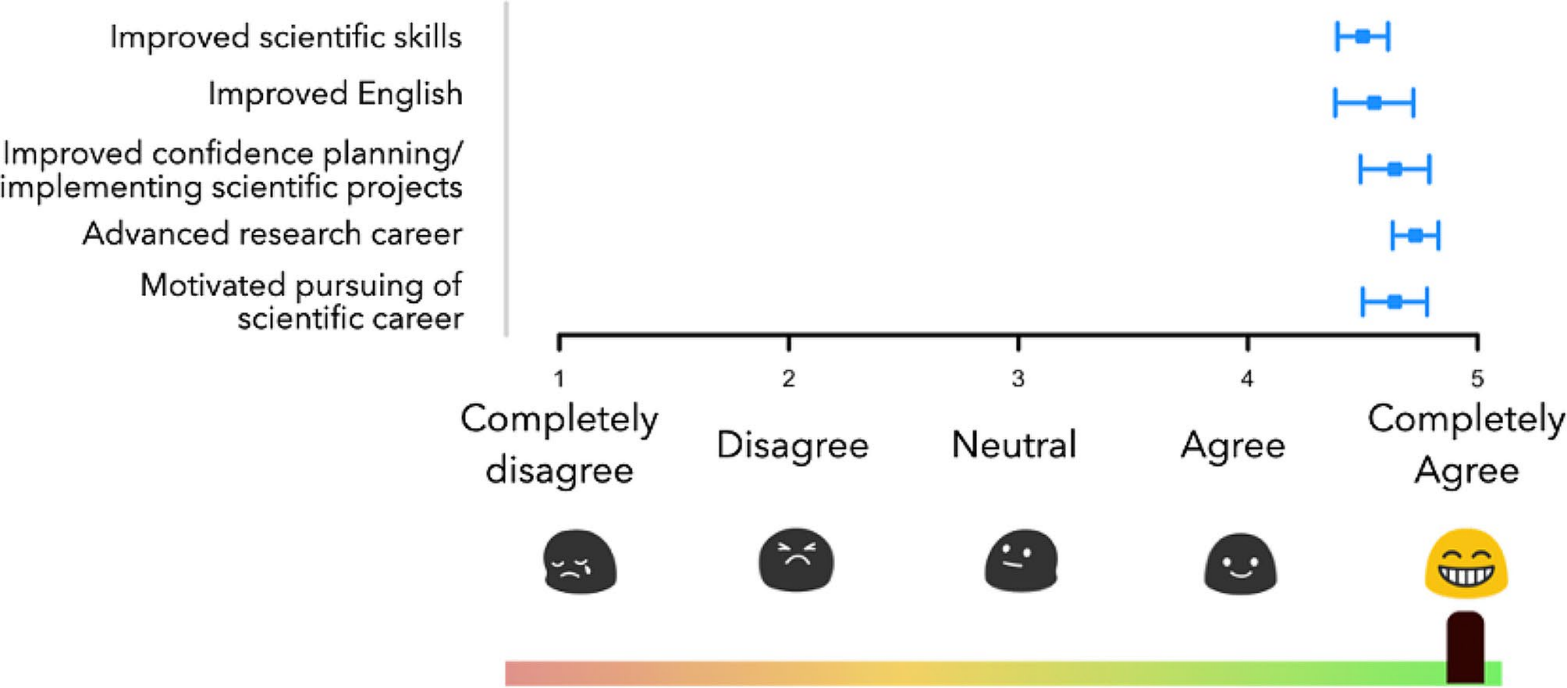
Overall program evaluation and its learning benefits for research skills. Error bars represent the standard error

The international virtual education program was highly appreciated by students and evaluated with 4.38 out of 5 stars (*SD* = 0.67). In particular, the items relating to the improvement of scientific and research skills were rated at very high levels (see Fig. 3). While the expectations of the students were largely met (*M* = 4.29; *SD* = 0.85), participants noted that they did not participate in as many courses as they had intended (*M* = 2.81; *SD* = 1.21). Reasons of 13 students who stated that they did not attend as many courses as planned were conflicts with their home university’s schedule (46%), their internship schedule (23%) or general timing/schedule issues (31%).

All courses were well appreciated by the participants. For an overview of the detailed ratings of each of the four course groups see Table 5. After the course work, students successfully completed the participation in vSEMERA with their poster presentations during the farewell week. Overall students’ performance, i.e., their poster designs, presentations as well as their answers to questions, was high with a mean of 51.79 out of maximum 65 points (*SD* = 4.13).

**Table 5:** Evaluation of the course groups

Course Group	Mean	SD	SE	*N* Questionnaire
Scientific Basics	4.62	0.56	0.10	29
Scientific Methods	4.00	1.03	0.26	16
Publications	4.32	0.93	0.15	38
Keynotes	4.00	0.89	0.37	6
Intercultural social program	4.50	0.71	0.50	2
Virtual/live poster walk	4.50	0.58	0.29	4
Farewell week	4.50	0.81	0.16	26

The high students’ performance and their enthusiasm was similarly mirrored in their overall evaluation of the program. In the following, an overview of the aspects that were highly appreciated is provided through personal statements made by the students. That is, participants highly appreciated the quality of the program, specific courses, and the enthusiasm of teachers as well as the social program. In the words of the students, within the open comment section of the questionnaire, that reads as follows:

“The quality and excellence of the professors that gave the lectures. In my home university I wouldn’t have access to these types of professionals so immersed in research.”

“I made friends during the program.” // “We got to know new people from different countries.”// “It was awesome get to know new people from different countries and to work with them through the different tasks during classes.” // “The social program for me was one of the best parts of the program.”

“I liked the final [poster] presentations. Because it was evidence of all the hard work the students did in the course.”

While the overall program received high appreciation, students suggested in the open comment sections to improve program specific organizational issues as well as the structure of the vSEMERA campus. Several students mentioned that the classes were rather time-consuming and not always compatible with their home university schedule and recommended offering the course content asynchronously to allow access at their convenience. These aspects were considered when organizing the following vSEMERA (now SEMERA) editions.

## Discussion

The current study evaluated the first edition of the multi-institutional and interdisciplinary IVE program vSEMERA. IVE programs in general are a promising avenue for expanding the horizons of students in the field of digital medical education. These programs connect students with international peers and perspectives, offering exciting opportunities for growth and learning. The IVE program vSEMERA was highly appreciated by the students and, according to students’ subjective experience, indeed presents a promising tool to improve medical students’ knowledge in the field of scientific skills. Notably, while the response rate of the questionnaires in the current study was low to moderate, the verbal feedback from the students matched the picture obtained through the questionnaires. Due to the COVID-19 pandemic, vSEMERA not only offered students an online training platform for their scientific skills, but also enabled them to connect virtually across the globe despite the pandemic. In addition to the general, virtual meeting-based character of the program, it was also the social program that benefited the international social exchange between the participants and counteracted the isolation of the learner. While the pandemic generally led to an increase in virtual tools [13], vSEMERA (now SEMERA) is continued to be offered to students around the world even after the pandemic due to the high appreciation by the students.

It should be noted, however, that the program’s successful implementation and continuous availability owe a great deal to the dedication and passion of the lecturers that, next to their duties at their home universities, made and make this program possible. A program like vSEMERA, while technologically forward and potentially enriching for students, comes with significant challenges, especially regarding teacher availability, their willingness to participate, and the logistical demands inherent to organizing such initiatives. Foremost among these is the issue of teacher availability. The varying time zones, commitments, and academic calendars can make it difficult to synchronize educators and students from diverse regions. In addition, the issue of willingness arises, as not all educators may possess technological skills or feel at ease in a virtual teaching setting. Likewise, it cannot be assumed that all students possess the necessary technical abilities or are comfortable with a virtual teaching environment. This potential limitation could restrict the number of teachers and students who can participate in such a setting. While technological skills can be developed, a key organizational challenge remains. Setting up and organizing an IVE program like vSEMERA demands robust technological infrastructure, effective communication channels, curriculum alignment, and, often, a significant amount of administrative support. These factors, combined, can introduce substantial costs, both tangible and intangible, to an institution seeking to implement a virtual exchange program. It’s crucial to weigh these challenges against the potential benefits, keeping in mind the end goal of delivering a comprehensive and enriching educational experience [26].

The following SEMERA editions will continue to maintain the positive aspects of the program and adapt the program based on student suggestions. This means that the research skills courses will continue to make up a large part of the program and maintain their high quality, and that the social program and thus the avoidance of learner isolation will continue to play an important role in the program. To ease participation across various time zones, given that students were especially commenting on the fact that they were unable to attend as many courses as they intended due to scheduling conflicts, the next vSEMERA editions from 2022 onwards (now SEMERA) were and will be conducted exclusively with asynchronous, virtual teaching formats. To keep the participants connected through the future asynchronous format, additional group work is added in synchronous format. Evaluations of the upcoming SEMERA editions will be conducted to assess whether similar results can be obtained and to control whether the feedback-informed changes made to the program can even further enhance the training of research skills. Notably, while learning outcomes in the current edition were only evaluated based on self-reports as well as self-directed knowledge tests, the future editions of the program will also assess students’ performance more directly. This will be done by tasks specified by the lectures of the courses, that are mandatory for the successful completion of the module. Lecturers provide feedback on students’ performance. Additionally, students must fulfil two major milestones (write a short systematic review and prepare and present a poster) that were rated by teachers of the program.

## Conclusion

After the successful launch of vSEMERA and extensive and overall, very positive feedback from participants, lecturers and cooperation partners, this program will be continued and further developed. vSEMERA is a great example of the potential of combining IVE, e-mobility, and scientific skills in health education. Yet, large-scale evaluations are needed to further guide the development of future e-learning and training.

## Data Availability

The datasets used and/or analyzed during the current study are available from the corresponding author on reasonable request.
